# Dietary arachidonate in milk replacer triggers dual benefits of PGE_2_ signaling in LPS-challenged piglet alveolar macrophages

**DOI:** 10.1186/s40104-019-0321-1

**Published:** 2019-02-15

**Authors:** Kathleen R. Walter, Xi Lin, Sheila K. Jacobi, Tobias Käser, Debora Esposito, Jack Odle

**Affiliations:** 1 Department of Animal Science, Plants for Human Health Institute, North Carolina State University, Kannapolis, North Carolina USA; 2 Department of Animal Science, North Carolina State University, Raleigh, North Carolina USA; 3 Department of Animal Science, Ohio State University, Columbus, Ohio USA; 4 Department of Population Health and Pathobiology, College of Veterinary Medicine, North Carolina State University, Raleigh, North Carolina USA

**Keywords:** Arachidonic acid, Cyclooxygenase, Eicosanoid, Eicosapentaenoic acid, Inflammation, Lipid mediator class switch, LPS, Lipoxin, Porcine alveolar macrophage

## Abstract

**Background:**

Respiratory infections challenge the swine industry, despite common medicinal practices. The dual signaling nature of PGE_2_ (supporting both inflammation and resolution) makes it a potent regulator of immune cell function. Therefore, the use of dietary long chain n-6 PUFA to enhance PGE_2_ effects merits investigation.

**Methods:**

Day-old pigs (*n* = 60) were allotted to one of three dietary groups for 21 d (*n* = 20/diet), and received either a control diet (CON, arachidonate = 0.5% of total fatty acids), an arachidonate (ARA)-enriched diet (LC n-6, ARA = 2.2%), or an eicosapentaenoic (EPA)-enriched diet (LC n-3, EPA = 3.0%). Alveolar macrophages and lung parenchymal tissue were collected for fatty acid analysis. Isolated alveolar macrophages were stimulated with LPS in situ for 24 h, and mRNA was isolated to assess markers associated with inflammation and eicosanoid production. Culture media were collected to assess PGE_2_ secretion. Oxidative burst in macrophages was measured by: 1) oxygen consumption and extracellular acidification (via Seahorse), 2) cytoplasmic oxidation and 3) nitric oxide production following 4, 18, and 24 h of LPS stimulation.

**Results:**

Concentration of ARA (% of fatty acids, *w*/*w*) in macrophages from pigs fed LC n-6 was 86% higher than CON and 18% lower in pigs fed LC n-3 (*P* < 0.01). Following LPS stimulation, abundance of *COX-2* and *TNF-α* mRNA (*P* <  0.0001), and PGE_2_ secretion (*P* < 0. 01) were higher in LC n-6 PAM vs. CON. However, *ALOX5* abundance was 1.6-fold lower than CON. Macrophages from CON and LC n-6 groups were 4-fold higher in *ALOX12/15* abundance (*P* < 0.0001) compared to LC n-3. Oxygen consumption and extracellular acidification rates increased over 4 h following LPS stimulation (*P* < 0.05) regardless of treatment. Similarly, increases in cytoplasmic oxidation (*P* < 0.001) and nitric oxide production (*P* <  0.002) were observed after 18 h of LPS stimulation but were unaffected by diet.

**Conclusions:**

We infer that enriching diets with arachidonic acid may be an effective means to enhance a stronger innate immunologic response to respiratory challenges in neonatal pigs. However, further work is needed to examine long-term safety, clinical efficacy and economic viability.

**Electronic supplementary material:**

The online version of this article (10.1186/s40104-019-0321-1) contains supplementary material, which is available to authorized users.

## Background

Respiratory infections in swine result in severe economic loss despite the wide spread use of vaccines and antibiotics [1]. Gram-negative bacteria are some of the common bacterial agents that afflict swine herds as either the primary infectious agent or as an opportunistic secondary infectious agent [1, 2]. Vaccines and antibiotics are routine practice however, the type of bacteria and timing of treatment can impact use and efficacy [1, 3]. In neonates, early life protection stems partially from passive immunity through the sow’s colostrum although the transfer of maternal antibodies can be limiting. Antibodies against certain Gram-negative bacteria can deplete in piglets within 1–4 weeks of life [3, 4]. The other stem of early life protection is the neonate’s innate immune system, as its adaptive immune system has not fully matured [4]. As such, neonates are at a much higher risk for infection. During this critical time, it is imperative to explore alternative means to enhance a stronger, well-balanced immunological response to respiratory infections.

Nutritional status, especially early in life, is a key component in immune system development, proper defenses, and susceptibility to infections. Dietary long chain polyunsaturated fatty acids (LC-PUFA), like arachidonic acid (ARA, n-6) and eicosapentaenoic acid (EPA, n-3), can modify the fatty acid composition of immune cells and their function directly through incorporation in to the phospholipid membrane and as free fatty acids acting indirectly as secondary messengers [5–7]. These LC-PUFA have immunomodulatory effects which can alter eicosanoid production and subsequent inflammatory responses in immune cells [8–10]. Arachidonic acid serves as a precursor for potent eicosanoids, like PGE_2_, which has the ability to promote pro-inflammatory signaling at the onset of an acute challenge. A lesser explored function of PGE_2_ is its ability to hinder initial pro-inflammatory signaling and promote anti-inflammatory and pro-resolving cascades, a mechanism referred to as “lipid mediator class switching” [11, 12]. The pro-inflammatory roles of ARA and anti-inflammatory roles of EPA have been extensively reviewed by Calder [13]. The involvement of PGE_2_ in lipid mediator class switching has been evaluated [14–16] and thoroughly reviewed by Serhan and colleagues [17–21]. Few studies focus on the dual role of PGE_2_, derived from ARA, and the potential benefits stemmed from this eicosanoid during acute health challenges. Studies are particularly sparse in swine.

Dietary supplementation with EPA is best known for its anti-inflammatory benefits. However, in instances where inflammation is essential for controlling and eradicating invading pathogens, increasing supplementation with EPA or other long chain n-3 PUFA could dampen an appropriate immunological response to respiratory pathogens. Macrophages enriched with n-3 PUFA demonstrate decreased phagocytosis [22], production of reactive oxygen species [23], ARA-derived eicosanoids [8, 9, 24, 25], and pro-inflammatory cytokine production [24, 26, 27]. There have been some studies regarding the effects of n-3 and n-6 PUFA modulating the immune response of respiratory pathogens in rodents. For instance, supplementation with long chain n-3 PUFA results in decreased bacterial clearance and survival to *Listeria monocytogenes* [28, 29] and *Mycobacterium tuberculosis* [30].

There are few studies regarding the effects of PUFA supplementation and respiratory health in swine, particularly involving the use of dietary long chain n-6 PUFA. Studies evaluating dietary n-6 PUFA predominantly involve dietary sources containing the precursor for ARA, linoleic acid. Studies have demonstrated the conversion of ARA, EPA, and DHA from their precursors linoleic acid and alpha-linoleic acid are low and supplementation with preformed LC-PUFA can be beneficial [31–35]. Very few studies, particularly for the swine industry, utilize preformed ARA sources. This study is the first to evaluate the effects of preformed long chain n-6 PUFA supplementation on the innate immune response to respiratory pathogens in neonatal pigs. Our aim was to determine if supplemental long chain n-6 PUFA in milk replacer-fed pigs could improve the innate immune response of alveolar macrophages (PAM) following *in situ* LPS stimulation. Given the duality of PGE_2_ signaling of both pro- and anti-inflammatory mechanisms, we hypothesized that a heightened but balanced immune response would dampen untoward cellular damage.

## Methods

### Piglets and experimental dietary treatments

All animal protocols were approved by the Institutional Animal Care and Use Committee of North Carolina State University. Animals were managed as previously described [36, 37]. Briefly, colostrum-fed piglets were acquired 24 h after birth and housed individually. Piglets (*n* = 60) were allotted to dietary treatment groups (*n* = 20/treatment) completely at random without regard to litter of origin, body weight or gender. The pigs used per litter ranged from 2 to 8. Pigs were allotted to a milk replacer diet (Table 1) supplemented with LC-PUFA containing either 0.5% ARA (ARASCO Oil, DSM Nutritional Products, Inc. Product # 5015002044) of total fatty acids (control (CON)), 2.2% ARA (LC n-6, ARASCO Oil) or 3.0% EPA (LC n-3) of total fatty acids (Table 2) for 21 d. The 0.5% ARA supplementation in CON diet satisfies the recommended level for infant formula [38]. The LC n-6 and n-3 PUFA diets were patterned after previous work in our laboratory [36, 37]. The LC n-6 diet was supplied with a higher concentration of ARA (2.2% of total fatty acids) to prophylactically enrich the fatty acid content of PAM with ARA. The LC n-3 diet was our negative control, and was supplemented to be isocaloric to the LC n-6 and CON diets with EPA-enriched fish oil (MegOil, DSM Nutritional Products, Inc. Product # 5015261). This provided 3.0% EPA of total fatty acids to the diet. The MegOil used for the LC n-3 diet contained a higher level of DHA and therefore was not further supplemented with the DHASCO oil (DSM Nutrition Products, Inc. Product # 5013658044). Vitamin E fortification was uniform across diets (155 IU/kg), and an antioxidant (TBHQ) was added at 0.1 g/kg. At the end of the trial, pigs were humanely euthanized by exsanguination under isoflurane anesthesia.

**Table 1:** Composition of milk replacer diet fed to piglets

Ingredient, g/kg	CON	LC n-6	LC n-3
Whey	444.6	444.6	444.6
Edible lard	44.0	44.0	44.0
Whey protein concentrate	101.4	101.4	101.4
Delactosed whey	51.4	51.4	51.4
Na casienate	112.5	112.5	112.5
Dicalcium phosphate	22.1	22.1	22.1
Other^b^	24.1	24.1	24.1
Fat blend supplement			
ARASCO^a^	3.0	14.8	0.0
DHASCO^a^	1.8	1.8	0.0
Coconut (Hydrogenated)	54.3	54.3	54.3
Soy oil	65.5	64.2	65.0
Sunflower, high oleic	65.3	54.8	55.8
EPA MegOil^a^	0.0	0.0	14.8
Tween80	10.0	10.0	10.0
TBHQ	0.1	0.1	0.1
Total	1000.0	1000.0	1000.0
Calculated composition			
Energy, kcal/g	5.2	5.2	5.2
Crude protein, %	25.0	25.0	25.0
Crude fat, %	26.0	26.0	26.0
Lactose, %	34.5	34.5	34.5

^a^Courtesy of DSM Nutritional Products

^b^Other, g/kg diet: mineral premix, 6.3; *D*/*L*-methionine, 6.2; potassium sorbate, 5.6; *L*-lysine HCl, 4.5; calcium chloride, 4.1; vitamin premix, 1.4; flavor additive, 0.6; emulsifier, 0.6; flow agent, 0.5; tetrasodium pyrophosphate, 0.3; antioxidant 0.01; Milk Specialties, Eden Prairie, MN 55344

**Table 2:** Analyzed fatty acid composition of milk replacer fed to piglets (g/100 g, fatty acid)^a^

Diet	CON	LC n-6	LC n-3
Fatty acid			
6:0	0.01	0.03	0.02
8:0	0.45	0.52	0.89
10:0	1.24	1.42	1.63
12:0	10.91	12.03	13.67
14:0	5.36	5.54	5.68
15:0	0.09	0.06	0.07
16:0	13.78	14.25	11.83
16:1	0.20	0.06	0.09
17:1	0.00	0.00	0.10
18:0	4.52	7.08	6.26
18:1	37.76	33.92	32.70
18:2 (n-6)	20.55	19.00	19.44
18:3 (n-6)	0.25	0.20	0.07
18:3 (n-3)	3.01	2.54	2.83
20:0	0.24	0.17	0.19
20:1	0.17	0.23	0.31
20:2	0.14	0.12	0.10
20:4 (n-6)	0.53	2.22	0.20
20:3 (n-3)	0.08	0.15	0.04
20:5 (n-3)	0.00	0.00	3.03
22:0	0.22	0.20	0.29
22:6 (n-3)	0.21	0.18	0.00
24:0	0.08	0.08	0.41
Total	100	100	100
SFA	37.10	41.55	40.80
MUFA	38.12	34.21	33.21
PUFA	24.78	24.42	26.13
n-3 FA	3.30	2.87	6.31
n-6 FA	21.48	21.54	19.82
n-3: n-6	0.15	0.13	0.32

^a^Data are expressed as weight % of total identified fatty acids. These fats represented 26% of the milk replacer dry matter as described in Table 1. *MUFA* monounsaturated fatty acid, *PUFA* polyunsaturated fatty acid, *SFA* saturated fatty acid

### Sample collection

Whole blood was collected via jugular venipuncture prior to anesthesia. Whole blood was collected in EDTA-treated tubes and blood for serum was collected in untreated tubes. Whole blood and serum were prepared and utilized the same day for clinical analyses of blood chemistry panels and complete blood cell counts by a commercial auto analyzer (Veterinary SuperChem/CBC, Antech Diagnostics) to assess general clinical health status of the pigs. Alveolar macrophages were isolated via bronchoalveolar lavage using Hanks’ Balanced Salt Solution as previously reported [39, 40]. Cells were centrifuged and pellets were re-suspended in freezing medium containing 70% RPMI-1640 media, 20% heat-inactivated fetal bovine serum (HI-FBS), and 10% dimethyl sulfoxide at concentrations of 2 × 10^7^ cells/mL. Cells were frozen in liquid nitrogen for subsequent cell culture. Lung tissue samples were obtained following bronchoalveolar lavage, snap-frozen in liquid nitrogen and stored at − 80 °C for subsequent fatty acid analysis.

### Validation and characterization of PAM isolation procedure

Cells isolated from lungs were characterized by flow cytometry. Lung cells were stained in 96-well round bottom plates (Thermo Fisher). To confirm the isolation of PAM, cells were stained for CD14, CD163 and CD172A. For the characterization of co-isolated lymphocytes, lung cells were stained for the T-cell marker CD3, CD8α to identify CD3^−^CD8α^+^ NK cells, and the pan-B cell marker CD21a (Table 3). Live/Dead discrimination (LIVE/DEAD® Fixable NearIR Dead Cell Stain Kit, ThermoFisher) confirmed that for all analyzed samples, over 97% of cells were alive in the PAM gate and over 95% in the lymphocyte gate (data not shown). Cells were analyzed on a BD LSR II (BD Biosciences).

**Table 3:** Staining reagents used in flow cytometry of immune cells isolated from lungs of milk replacer-fed pigs

Antigen	Clone	Isotype	Fluorochrome	Labeling strategy	Primary antibody source	2^nd^ Antibody source
Immunotyping of myeloid cells						
CD14	TüK4	IgG2a	FITC	Directly conjugated	ThermoFisher	–
CD163	2A10/11	IgG1	PE	Directly conjugated	ThermoFisher	–
CD172A	74–22-15A	IgG2b	Alexa 647	Secondary antibody	BEI Resources	Southern Biotech
Immunotyping of lymphocytes						
CD3	PPT3	IgG1	Alexa 488	Secondary antibody^a^	BIO RAD	Southern Biotech
CD8α	76–2-11	IgG2a	Alexa 647	Secondary antibody	BEI Resources	Southern Biotech
CD21a	BB6-11C9.6	IgG1	PE-Cy5.5	Streptavidin-biotin	Novus Biologicals	Southern Biotech

^a^Free binding sites of secondary antibody were blocked by ChromePure Mouse IgG (Jackson Immuno Research, West Grove, PA) prior to anti-CD21a staining

### Fatty acid analysis

Composition of total fatty acids were determined in lung tissue, PAM, and milk sample composites collected throughout the duration of the trial. Porcine alveolar macrophages were thawed on ice, and fatty acids were subjected to direct-methylation [41] with some modification. Cells were washed in 1× PBS and centrifuged at 180 × *g* at room temperature for 5 min. Supernatant was removed and 100 mg of cells were transferred to a 20-mL Teflon-lined, screw-capped tube. One mL of methanol and 3 mL of 3 mol/L methanolic-HCl were added. Tubes were capped tightly and refluxed in a 95 °C-water bath for 1 h. Eight mL of 0.88% NaCl (*wt*: *vol*) and 3 mL of hexane were added to each sample, vortexed, and centrifuged at 1330 × *g* for 15 min at 4 °C. After centrifugation, the top layer was transferred to a 1.5-mL vial and evaporated to dry under N_2_. Fatty acids from milk and tissue samples were extracted and saponified as previously described [42], with some modification. One hundred mg of tissue sample was homogenized in 1 mL sterile water. Samples were centrifuged at 1330 × *g* at 4 °C. Fatty acid methyl esters were dissolved in 25 μL hexane and analyzed on a weight percent basis of total fatty acids using gas chromatography-mass spectrometry (GC-MS) as previously described [42].

### Cell culture and mRNA analysis

Porcine alveolar macrophages from all dietary treatment groups were cultured in RPMI 1640 media supplemented with L-glutamine, penicillin (100 U/mL), streptomycin (100 μg/mL), fungizone (4 μg/mL), gentamycin (50 μg/mL), and 10% HI-FBS. Cells were thawed in a 37 °C water bath, washed with warmed culture media and centrifuged at 180 × *g* at room temperature for 10 min [43]. Supernatant was removed, cells were re-suspended in warmed media and seeded as a composite of six pigs per dietary treatment on 6-well plates at a density of 3 × 10^6^ cells/mL in triplicate. Cells were either stimulated with 10 ng/mL of LPS (*Escherichia coli* O111:B4) or not stimulated (basal), and maintained at a 37 °C humidified incubator with 5% CO_2_ for 24 h. Selection of dosage and timeline for LPS stimulation were based on a preliminary study that examined the dose and time dependence of PGE_2_ production at 0, 3, 6, 12, 24, and 48 h following stimulation with 10, 100, and 1000 ng/mL LPS from the LC n-6 treatment group (data not shown). Cells were collected in TRIzol Reagent (Ambion) and triplicate wells were pooled. Total RNA was purified according to manufacturer’s instructions with the modification that RNA precipitation occurred overnight at − 80 °C. Complementary DNA synthesis was carried out using a High Capacity cDNA Reverse Transcription kit (Applied Biosystems) following manufacturer’s instructions. Primers for pig ribosomal proteins L4 (*RPL4)* and L9 *(RPL9),* tyrosine 3-monooxygenase/tryptophan 5-monooxygenase activation protein zeta polypeptide (*YWHAZ),* succinate dehydrogenase complex subunit A (*SDHA*)*,* toll-like receptor 4 *(TLR-4), COX-1, COX-2,* lipoxygenase 5 (*ALOX5)* and 12/15 (*ALOX12/15*)*,* tumor necrosis factor alpha (*TNF-α*), interleukin 6 (*IL-6*) and 10 (*IL-10*) (Table 4) were designed using primer-BLAST from the National Center for Biotechnology Information (NCBI) database. Primers were designed to span exon-exon junctions. Melt curve analysis was performed on all primers and samples to confirm the absence of primer dimers and existence of gene-specific peaks. Genes of interest were normalized to the geometric mean of the housekeeping genes *RPL4, RPL9, YWHAZ,* and *SDHA*. Measurement of mRNA abundance was performed by qRT-PCR and the 2^-ΔΔCt^ method as previously described [44–46]. All samples were run in duplicate. Relative expression was normalized to the basal CON treatment.

**Table 4:** Sense and anti-sense primer sequences (5′→3′) used in qRT-PCR analysis

Target	GenBank accession	Amplicon size, bp	Primer sequence
*RPL4*	XM_005659862.3	110	F: TTCAAGGCTCCCATTCGACC
			R: GCACTGGTTTGATGACCTGC
*RPL9*	XM_013978597.2	106	F: GGGTTGACAAATGGTGGGGA
			R: TTGTAACGGAAGCCCAGTGT
*SDHA*	XM_021076931.1	106	F: TTGCGAACGGAACCATAAGGA
			R: CAGCCTTCCTGTAACACGCT
*YWHAZ*	XM_001927228.7	105	F: AAGAAGGGGATTGTGGATCAGTC
			R: AAGGGCCAGACCCAATCTGA
*TLR-4*	NM_001113039.2	112	F: CGTGCAGGTGGTTCCTAACA
			R: CAGGTAGTTAAAGCTCAGGTCCA
*COX-1*	XM_001926129.6	116	F: ACACGGCACACGACTACATC
			R: CTTCTTCCCTTTGGTCCCCAT
*COX-2*	NM_214321.1	90	F: ATGGGTGTGAAAGGGAGGAAAG
			R: CTGGGGATCAGGGATGAACTT
*5-LOX*	XM_021072736.1	103	F: ATGCCAAATGCCACAGGGAT
			R: ATGAACAGGTTCTCCATCGCTT
*12/15-LOX* [90]	NM_213931	97	F: CAGGCTTGGTGTCGAGAGTT
			R: AGTGGCAGAGCTGTTCCTTG
*TNF-α*	NM_214022.1	94	F: CCCCAGAAGGAAGAGTTTCCA
			R: CGACGGGCTTATCTGAGGTTT
*IL-6*	NM_001252429.1	101	F: GACCCTGAGGCAAAAGGGAA
			R: TCCACTCGTTCTGTGACTGC
*IL-10* [91]	NM_214041.1	101	F: GACGTAATGCCGAAGGCAGA
			R: AGGGCAGAAATTGATGACAGCG

### PGE_2_ ELISA assay

Tissue culture media from all PAM were collected at time of RNA isolation. Media from triplicate wells were pooled and used to determine the concentration of PGE_2_. A competitive enzyme-linked immunosorbent assay (ELISA) kit specified for porcine PGE_2_ (Pierce) was utilized following manufacturer’s instructions. The assay range was 39.1–2500 pg/mL, with a minimum detectable dose of 13.4 pg/mL. Samples that did not fall within detectable range of this ELISA were diluted 50× in the same type of media in which they were cultured, as suggested by the manufacturer. All samples were assayed in duplicate. Inter- and intra-assay coefficient of variation were assessed to validate precision. The inter-assay was 3.25% and determined using a composite of 19 pigs from the CON group stimulated with 10 ng/mL LPS. The intra-assay was 2.62% and determined using values from the median point of the standard curve for each plate.

### Respiratory burst analysis

Occurrence of respiratory burst in PAM was determined via three independent assays following LPS stimulation. First, oxygen consumption rate (OCR) and extracellular acidification rate (ECAR) upon LPS stimulation were determined using an Extracellular Flux XF Analyzer (Seahorse Bioscience) as previously described using RAW 264.7 macrophages with low *n*-values by Grace et al. [47, 48]. Second, PAM were cultured and stimulated with 10 ng/mL LPS for 18 and 24 h, and oxidative stress was determined using a commercially available CellROX Orange Reagent following manufacturer’s instructions. Briefly, cells were seeded at a density of 3 × 10^5^ cells per well in a 96-well plate. Cells were cultured and stimulated with LPS following the same protocol as previously stated, with the exception of different time points. NucBlue Live ReadyProbes Reagent and CellROX Orange Reagent (Invitrogen) was added to tissue culture medium of plated cells at 1 drop/mL and a final concentration of 5 μmol/L respectively following LPS stimulation. Cells were incubated at 37 °C for 30 min. Media was removed and cells were washed three times with 1X PBS. Cells were imaged using an EVOS FL Auto Cell Imagining System (Life Technologies). Quantification of oxidative stress was measured at an excitation/emission of 545/565 nm using a BioTek Synergy H1 Multi-mode microplate reader (BioTek Instruments). Third, tissue culture media from all cultured PAM were collected at 18 and 24 h post LPS stimulation for measurement of nitrite concentration as an indication of nitric oxide (NO) production using the Greiss Reagent System (Promega) according to manufacturer’s instructions. Quantification for NO production was measured on a BioTek Synergy H1 Multi-mode microplate reader at an absorbance of 540 nm.

### Statistical analysis

Concentrations of fatty acids, serum metabolite, and blood cell count data were analyzed by a one-way ANOVA using the general linear model procedure of SAS (version 9.4) and the least significant differences multiple comparison test (SAS Institute). Data from gene expression, PGE_2_ concentration, oxidative stress and respiratory burst analysis were analyzed according to a two-way ANOVA for a 3 × 2 factorial design (diets +/− LPS) with the Tukey multiple comparison test. Residual analysis confirmed that ANOVA assumptions of normality and homogeneity of error variance were met. Data reported are least square means ± SEM. Statistical significance was declared when *P* <  0.05 and trends were noted when 0.05 < *P* < 0.1.

## Results

### Dietary supplementation and pig performance

Pigs averaged 1.87 ± 0.07 kg at the beginning of the trial and 6.64 ± 0.07 kg at end of the trial (data not shown). Diets were formulated to be isocaloric (Table 1) and total fatty acid composition was confirmed via GC-MS (Table 2). Feed intake and growth rates were unaffected by dietary treatments (*P* > 0.1; data not shown). All serum metabolites and blood cell counts (Table 5) fell within normal clinical ranges. Serum concentrations of urea nitrogen, cholesterol and mean corpuscular volume were lower in LC n-3 fed pigs (*P* < 0.05), while albumin was elevated (*P* < 0.001) compared to CON and LC n-6 fed pigs. Other serum enzymes, metabolites and blood cell counts did not differ between dietary treatment groups (*P >* 0.1).

**Table 5:** Serum metabolite and enzyme concentrations and whole-blood cell from milk replacer-fed pigs^1,2^

Diet	CON	LC n-6	LC n-3	SEM	*P*-value
Blood chemistry panel					
Total protein, g/dL	5.25	4.7	5	0.3	0.45
Albumin, g/dL	2.75^a^	2.63^a^	3.03^b^	0.05	0.002
Globulin, g/dL	2.5	2.08	1.98	0.28	0.40
Albumin/Globulin ratio	1.2	1.3	1.56	0.16	0.29
Aspartate transaminase, U/L	24.25	20.25	32	6.68	0.48
Alanine transaminase, U/L	27.75	21.75	23	3.85	0.53
Alkaline phosphatase, U/L	636	750	702	69.32	0.53
Gama-glutamyl transpeptidase, U/L	37.75	31.75	24.25	8.47	0.55
Total bilirubin, mg/dL	0.1	0.1	0.13	0.01	0.41
Urea nitrogen, mg/dL	3^a^	2.25^b^	2^bc^	0.14	0.002
Creatinine, mg/dL	0.73	0.73	0.7	0.06	0.95
Blood urea nitrogen/Creatinine ratio	4.25	3	3	0.36	0.06
Phosphorus, mg/dL	11.7	11.45	11.5	0.31	0.83
Glucose, mg/dL	137	156	137	7.37	0.18
Calcium, mg/dL	12.55	12.38	12.33	0.16	0.59
Magnesium, mEq/L	2.2	2.2	2.13	0.12	0.88
Sodium, mEq/L	139	136	137	1.08	0.29
Potassium, mEq/L	7.125	7.5	7.33	0.45	0.84
Sodium/Potassium ratio	19.75	18	19	1.32	0.66
Chloride, mEq/L	101	101	101	0.91	0.93
Cholesterol, mg/dL	107^a^	105^a^	87.25^b^	5.21	0.05
Triglycerides, mg/dL	148	114	88.75	20.78	0.18
Amylase, U/L	1781	1648.25	5030	1889	0.40
Creatine phosphokinase, U/L	515	366	333	96.32	0.40
Complete blood count					
White blood cells, 10^3^/μL	11.75	9.4	8.43	1.43	0.29
Red blood cells, 10^6^/μL	5.55	5.6	6.15	0.18	0.08
Hemoglobin, g/dL	10.1	9.95	10.8	0.34	0.22
Hematocrit, %	36.5	33.75	36.25	1.23	0.27
Mean corpuscular volume, fL	66.25^a^	60.25^b^	59^bc^	1.63	0.03
Mean corpuscular hemoglobin, pg	18.25	17.78	17.58	0.45	0.57
Mean corpuscular hemoglobin concentration, g/dL	27.65	29.63	29.75	0.74	0.14
Platelet count, 10^3^/μL	578	486.25	360.25	124.27	0.49
Neutrophils, %	34.75	37.5	39.35	6.02	0.87
Bands, μL	0	0	0	0	–
Lymphocytes, %	58.5	52.5	52.5	5.48	0.68
Monocytes, %	5.50	5.75	5.00	0.91	0.84
Eosinophils, %	1.25	4.25	3.25	1.65	0.46
Basophils, %	0	0	0	0	–

^1^Diets contained 0.5% ARA (CON), 2.2% ARA (LC n-6) or 3.0% EPA (LC n-3**)** of total fatty acids

^2^Values are least-square means and SEM, *n* = 4

^a, b, c^Means within a row lacking a common letter differ (*P* < 0.05)

### Phenotyping of isolated lung cells from bronchoalveolar lavage

Cells isolated via bronchoalveolar lavage could be distinguished into three subsets based on their forward and side scatter properties (FSC/SSC): dead cells (on average: 15.5%), PAM (on average: 76.4%), and lymphocytes (on average: 8.0%). Within the PAM scatter gate, cells isolated from all pigs demonstrated the characteristic expression pattern of PAM: CD14^low^CD163^+^CD172A^+^. Within the lymphocyte scatter gate, 18.3% were CD3^+^ T cells, 34.3% were CD21a^+^ B cells, and 1.8% were CD8α^+^ NK cells (Additional file 1).

### Lung and PAM fatty acid composition

Arachidonic acid (20:4n-6) concentration in lung parenchymal tissue was 1.4-fold higher (*P* < 0.0001) in pigs receiving the LC n-6 diet compared to pigs receiving CON diet for 21 d (Table 6). A similar enrichment pattern was observed in PAM (Table 6). Concentration of ARA was 2-fold higher in PAM from pigs receiving the LC n-6 diet compared to pigs receiving CON and LC n-3 diets (*P* < 0.01). The concentration of ARA was also higher in PAM than in lung parenchyma (*P* < 0.01). As the concentration of ARA increased, the concentrations of oleic acid (18:1) and linoleic acid (18:2n-6) tended to lower in both lung tissue and PAM. The proportions of 18:1, 18:2n-6 and 18:3n-3 (*P* < 0.01) were higher on average in parenchymal lung tissue than in PAM, while PAM were more highly enriched in 22:6n-3 by at least 90% (*P* < 0.04).

**Table 6:** Fatty acid composition of lung parenchymal tissue and porcine alveolar macrophages (PAM)^1–2^

Tissue	Lung				PAM			
Diet	CON	LC n-6	LC n-3	*P* - value	CON	LC n-6	LC n-3	*P* - value
Fatty acid, g/100 g FA								
14:0	1.62 ± 0.44	1.81 ± 0.38	2.04 ± 0.24	0.68	1.66 ± 0.19	1.53 ± 0.22	1.93 ± 0.22	0.45
16:0	31.38 ± 1.20	33.13 ± 1.04	33.45 ± 0.66	0.33	38.48 ± 3.31	33.62 ± 3.82	32.71 ± 3.82	0.47
16:1	0.66 ± 0.04 ^a^	0.50 ± 0.04 ^b^	0.50 ± 0.02 ^b^	0.01	0.82 ± 0.13	0.46 ± 0.15	0.74 ± 0.15	0.22
18:0	20.56 ± 1.29	19.01 ± 1.12	20.23 ± 0.71	0.59	24.31 ± 1.65	25.44 ± 1.90	21.38 ± 1.90	0.32
18:1	22.94 ± 1.02	21.85 ± 0.88	22.82 ± 0.56	0.61	14.43 ± 1.18	13.02 ± 1.34	17.71 ± 1.34	0.06
18:2	10.06 ± 0.54 ^a^	8.21 ± 0.47 ^b^	10.83 ± 0.30 ^a^	0.0002	5.76 ± 0.73^xy^	5.22 ± 0.84^x^	8.11 ± 0.84^y^	0.06
18:3 (n-6)	0.12 ± 0.03 ^a^	0.02 ± 0.03 ^b^	0.00 ± 0.02 ^b^	0.01	1.25 ± 0.19	1.48 ± 0.22	1.23 ± 0.22	0.67
18:3 (n-3)	0.22 ± 0.07	0.17 ± 0.06	0.16 ± 0.04	0.76	0.03 ± 0.04^x^	0.00 ± 0.04^x^	0.17 ± 0.04^y^	0.02
20:0	0.40 ± 0.09	0.45 ± 0.07	0.45 ± 0.05	0.86	0.58 ± 0.08	0.38 ± 0.09	0.55 ± 0.09	0.27
20:1	0.43 ± 0.05 ^a^	0.28 ± 0.04 ^b^	0.30 ± 0.03 ^b^	0.03	0.47 ± .09	0.23 ± 0.10	0.47 ± 0.10	0.16
20:2	0.7 ± 0.053^a^	0.56 ± 0.04 ^b^	0.50 ± 0.03 ^b^	0.001	0.73 ± 0.12	0.58 ± 0.14	0.34 ± 0.14	0.15
20:4 (n-6)	8.26 ± 0.60 ^a^	11.55 ± 0.52 ^b^	4.56 ± 0.33 ^c^	< 0.0001	7.66 ± 1.40^x^	14.22 ± 1.62^y^	6.27 ± 1.62^x^	0.01
20:3 (n-3)	0.62 ± 0.08	0.43 ± 0.07	0.60 ± 0.04	0.10	0.70 ± 0.11	0.67 ± 0.12	0.57 ± 0.12	0.74
20:5 (n-3)	0.00 ± 0.15^a^	0.00 ± 0.13^a^	1.53 ± 0.08^b^	< 0.0001	0.02 ± 0.28^x^	0.00 ± 0.33 ^x^	3.44 ± 0.33^y^	< 0.0001
22:0	0.68 ± 0.19	0.69 ± 0.16	0.85 ± 0.10	0.60	1.90 ± 0.18	1.81 ± 0.21	1.88 ± 0.20	0.95
22:1	0.60 ± 0.22	0.73 ± 0.19	0.56 ± 0.12	0.74	0.02 ± 0.05^x^	0.00 ± 0.06^x^	0.23 ± 0.06^y^	0.03
22:6 (n-3)	0.63 ± 0.11	0.63 ± 0.10	0.62 ± 0.06	0.99	1.19 ± 0.26^x^	1.34 ± 0.30^x^	2.27 ± 0.30^y^	0.04
Total	100	100	100		100	100	100	
MUFA	24.63 ± 1.11	23.36 ± 0.96	24.18 ± 0.61	0.67	15.73 ± 1.32	13.72 ± 1.50	19.15 ± 1.50	0.06
PUFA	20.65 ± 1.17	21.57 ± 1.02	18.80 ± 0.64	0.07	17.34 ± 2.23	23.50 ± 2.76	22.41 ± 2.76	0.21
n-3 FA	1.47 ± 0.31^a^	1.23 ± 0.27^a^	2.91 ± 0.17^b^	< 0.0001	1.94 ± 0.63^x^	2.01 ± 0.57^x^	6.45 ± 0.57^y^	< 0.0001
n-6 FA	19.18 ± 0.94^a^	20.34 ± 0.82^a^	15.89 ± 0.52^b^	< 0.0001	15.40 ± 2.02	21.48 ± 2.30	15.96 ± 2.30	0.13
n-3: n-6	0.08 ± 0.01^a^	0.06 ± 0.02^a^	0.18 ± 0.01^b^	< 0.0001	0.13 ± 0.01^x^	0.09 ± 0.02^x^	0.40 ± 0.02^y^	< 0.0001

^1^Values are least square means ± SEM. FA, fatty acid; MUFA, monounsaturated fatty acid; PUFA, polyunsaturated fatty acid

^2^Values represent weight % of total fatty acids

^a, b, c; x, y, z^Means within a row lacking a common letter differ (*P* < 0.05)

Pigs receiving the LC n-3 diet were more than 2-fold lower in 20:4n-6 content and 1.5-fold higher in 20:5n-3 enrichment in lung tissue (*P* < 0.0001) and PAM (*P* < 0.0001) compared to pigs receiving CON and LC n-6 diets. In PAM, pigs receiving the LC n-3 diet were 2-fold higher in 22:6n-3 (*P* < 0.04) compared to CON and LC n-6 groups.

### COX-2 mRNA abundance and PGE_2_ secretion

**Fig. 1:**
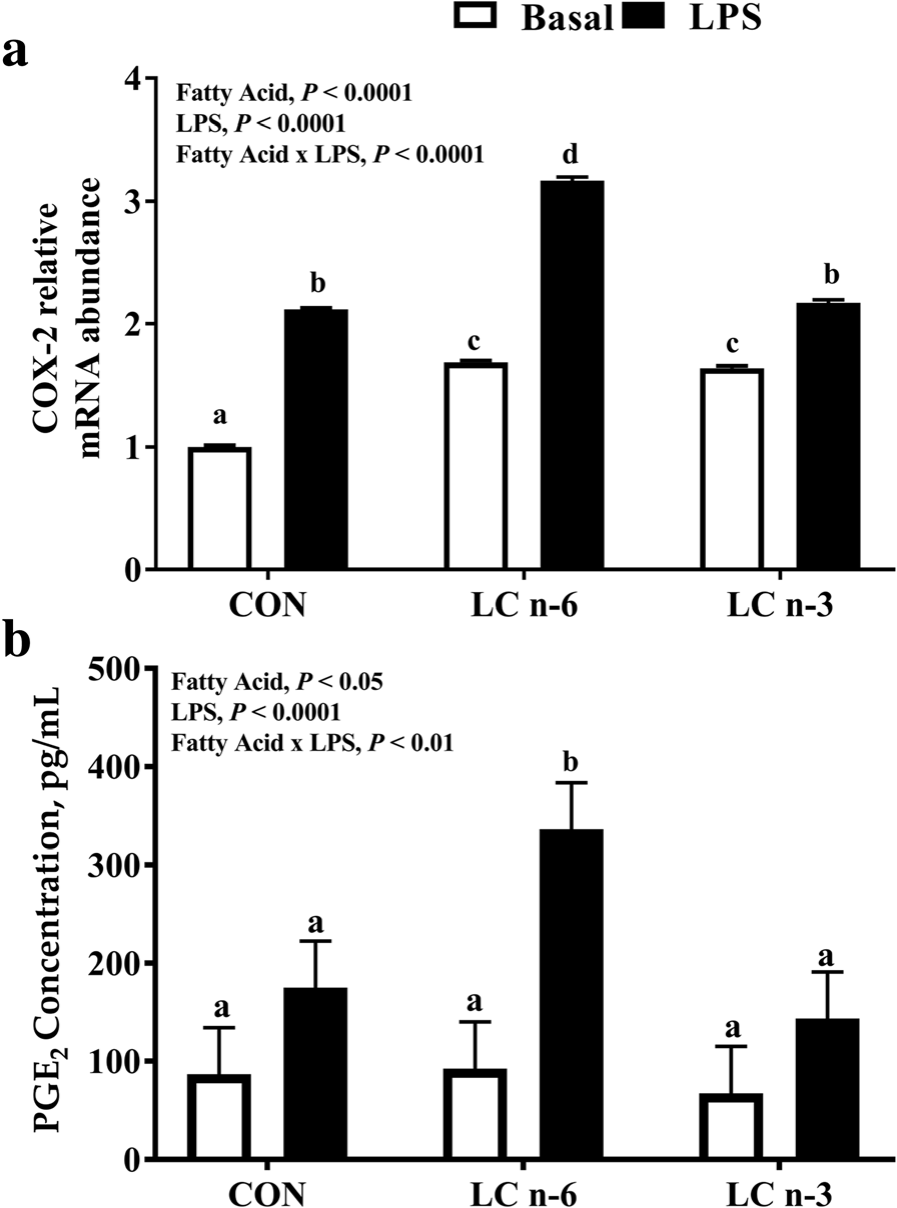
Dietary LC n-6 PUFA increases *COX-2* mRNA abundance and eicosanoid production in porcine alveolar macrophages. Relative expression level of (**a**) *COX-2* mRNA and (**b**) PGE_2_ production in alveolar macrophages isolated from piglets fed milk replacer with varying fatty acid composition. Measurements were made after 24 h of culture in absence (Basal) or presence of LPS. RNA values are mean fold changes relative to basal CON alveolar macrophages. Fatty acid effect, LPS effect and fatty acid-LPS interaction were evaluated. Values are represented as least square means ± SEM, (**a**) *n* = 12; (**b**) *n* = 20. Bars lacking a common letter differ

Following 24-h LPS stimulation, the relative mRNA abundance of *COX-2* was 4-fold higher in PAM for the LC n-6 dietary treatment group compared to the basal CON dietary group (*P* < 0.05). No detectable difference in *COX-2* mRNA abundance between the LC n-3 and CON groups were observed following LPS stimulation, however abundance was higher in LC n-3 basal compared to CON basal (Fig. 1a). Consistent with elevated *COX-2* mRNA abundance and ARA concentration, PGE_2_ concentration in PAM from the LC n-6 dietary treatment group was 2-fold higher following LPS stimulation compared to the basal CON dietary group (*P* < 0.0001). No detectable difference in PGE_2_ concentration was observed in PAM from the LC n-3 dietary group (Fig. 1b).

### Pro-inflammatory lipoxygenase and cytokine mRNA abundance

**Fig. 2:**
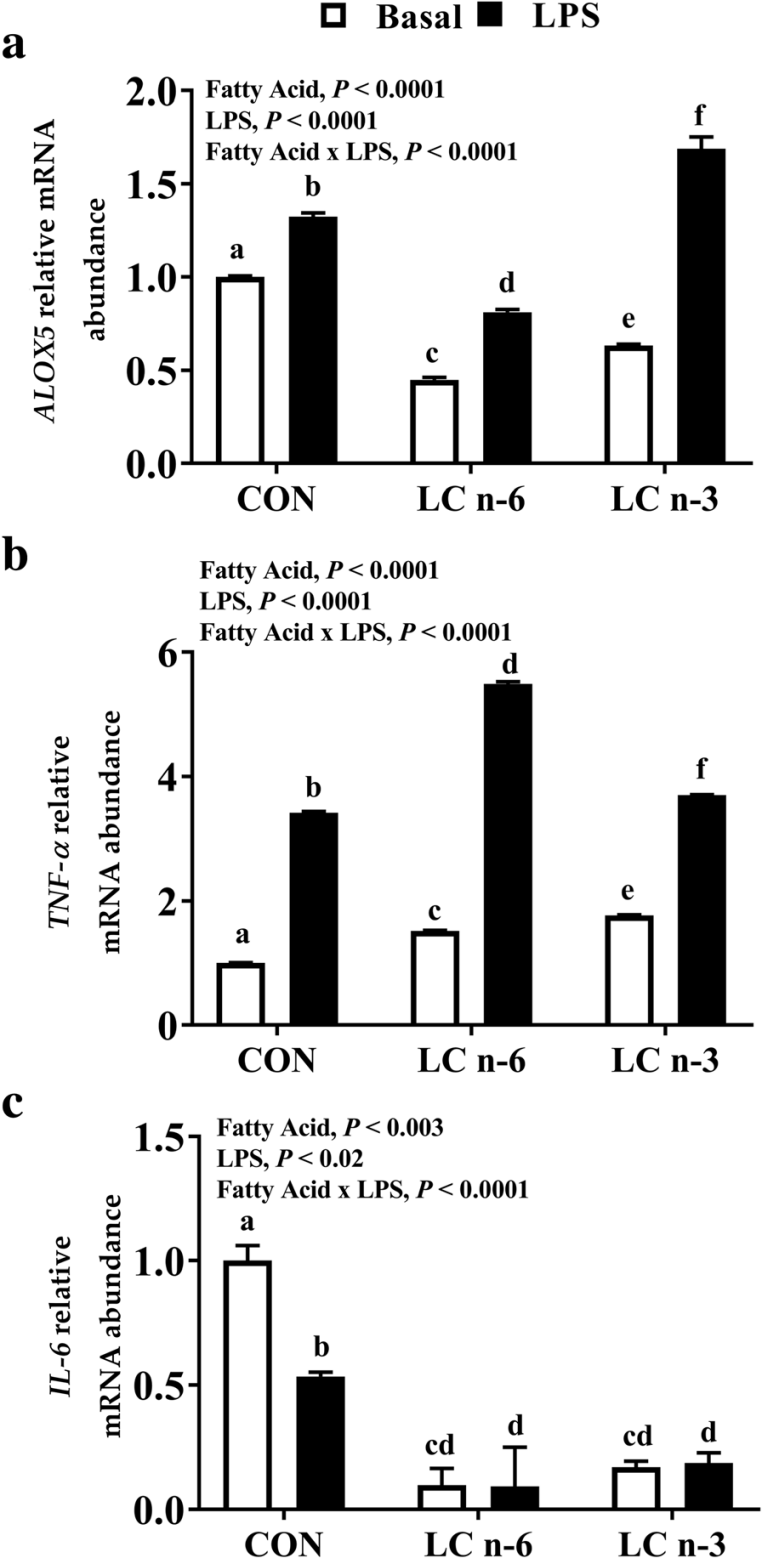
Dietary LC n-6 modifies lipoxygenase and cytokine mRNA abundance associated with a pro-inflammatory response in porcine alveolar macrophages. Relative mRNA abundance of (**a**) *ALOX-5*, (**b**) *TNF-α*, and (**c**) *IL-6* in alveolar macrophages from piglets fed milk replacer with varying fatty acid composition. Measurements were made after 24 h of culture in absence (Basal) or presence of LPS. Values are mean fold changes relative to basal CON alveolar macrophages. Fatty acid effect, LPS effect and fatty acid-LPS interaction were assessed. Values are represented as least square means ± SEM, *n* = 12. Bars lacking a common letter differ

In all dietary groups, differences in the relative mRNA abundance of Toll-like receptor 4 (*TLR-4*) and *COX-1* were not detected (data not shown). Treatment with LPS increased the abundance of *ALOX-5* mRNA in PAM from all dietary treatment groups by an average of 27% however, abundance in PAM from the LC n-6 fed pigs was 1.6-fold lower compared to CON and 2-fold lower compared to the LC n-3 treatment group (Fig. 2a). Additionally, *TNF-α* mRNA abundance was on average 4-fold higher following LPS-stimulation of PAM from the LC n-6 treatment group compared to CON and LC n-3 enriched alveolar macrophages (Fig. 2b). Differences in *IL-6* mRNA abundance were undetectable between the LC n-6 and LC n-3 groups, however CON alveolar macrophages were 47% lower following LPS stimulation (Fig. 2c).

### Anti-inflammatory lipoxygenase and cytokine mRNA abundance

**Fig. 3:**
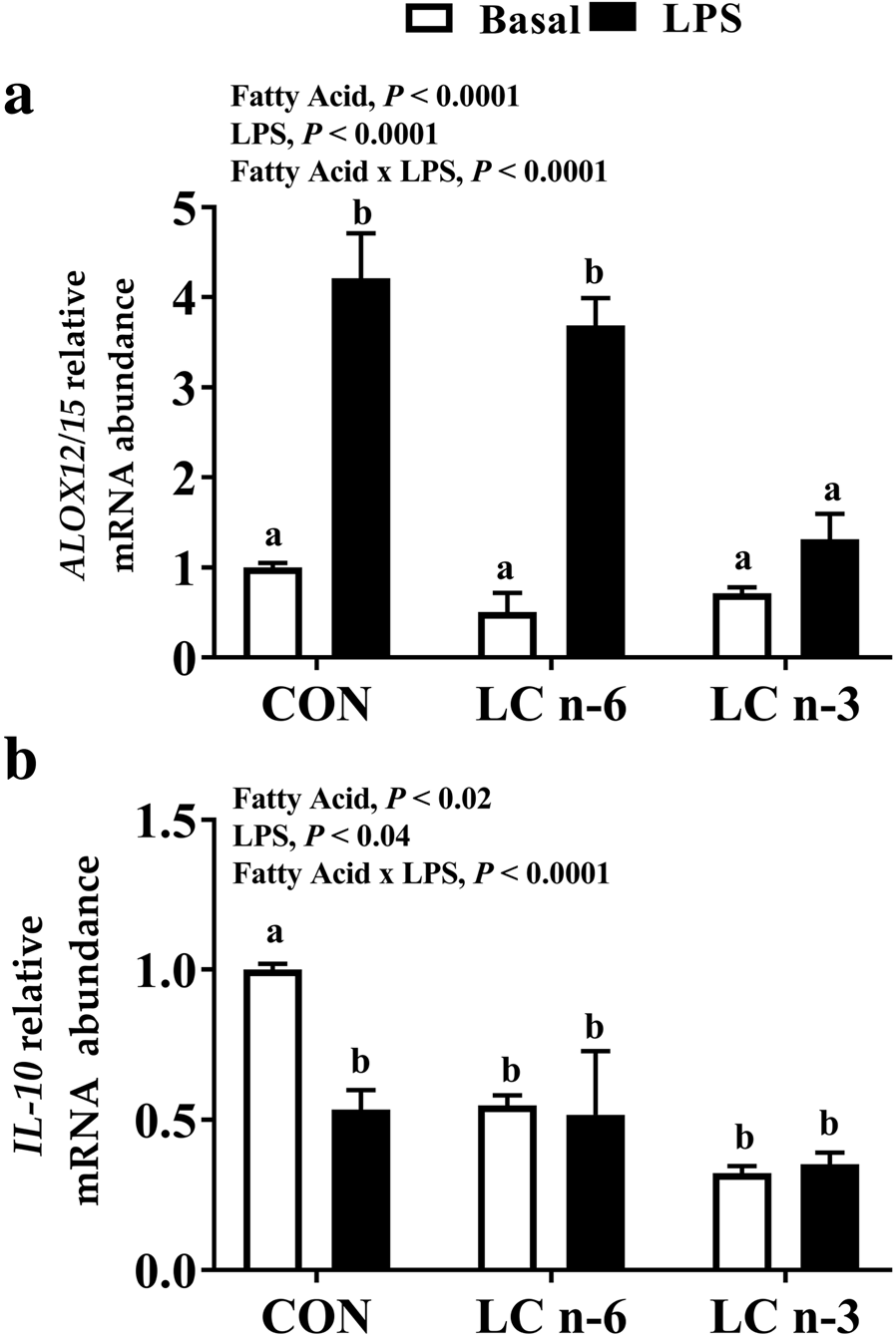
Long chain n-6 supplementation initiates initial stages of lipid-mediator class switching in porcine alveolar macrophages. Relative mRNA abundance of (**a**) *ALOX-12/15* and (**b**) *IL-10* in alveolar macrophages from piglets fed milk replacer with varying fatty acid composition. Measurements were made after 24 h of culture in absence (Basal) or presence of LPS. Values are mean fold changes relative to basal CON alveolar macrophages. Fatty acid effect, LPS effect and fatty acid-LPS interaction were assessed. Values represented as least square means ± SEM, *n* = 12. Bars lacking a common letter differ

Compared to CON basal and following LPS induction, alveolar macrophages from the CON and LC n-6 dietary treatment groups were 4-fold higher in *ALOX12/15* mRNA abundance, while no detectable difference was observed in macrophages from the LC n-3 treatment group (Fig. 3a). A significant difference in IL-10 abundance between CON basal and other dietary treatment groups prior to LPS stimulation was observed but not after (Fig. 3b).

### Oxidative burst and nitric oxide production

**Fig. 4:**
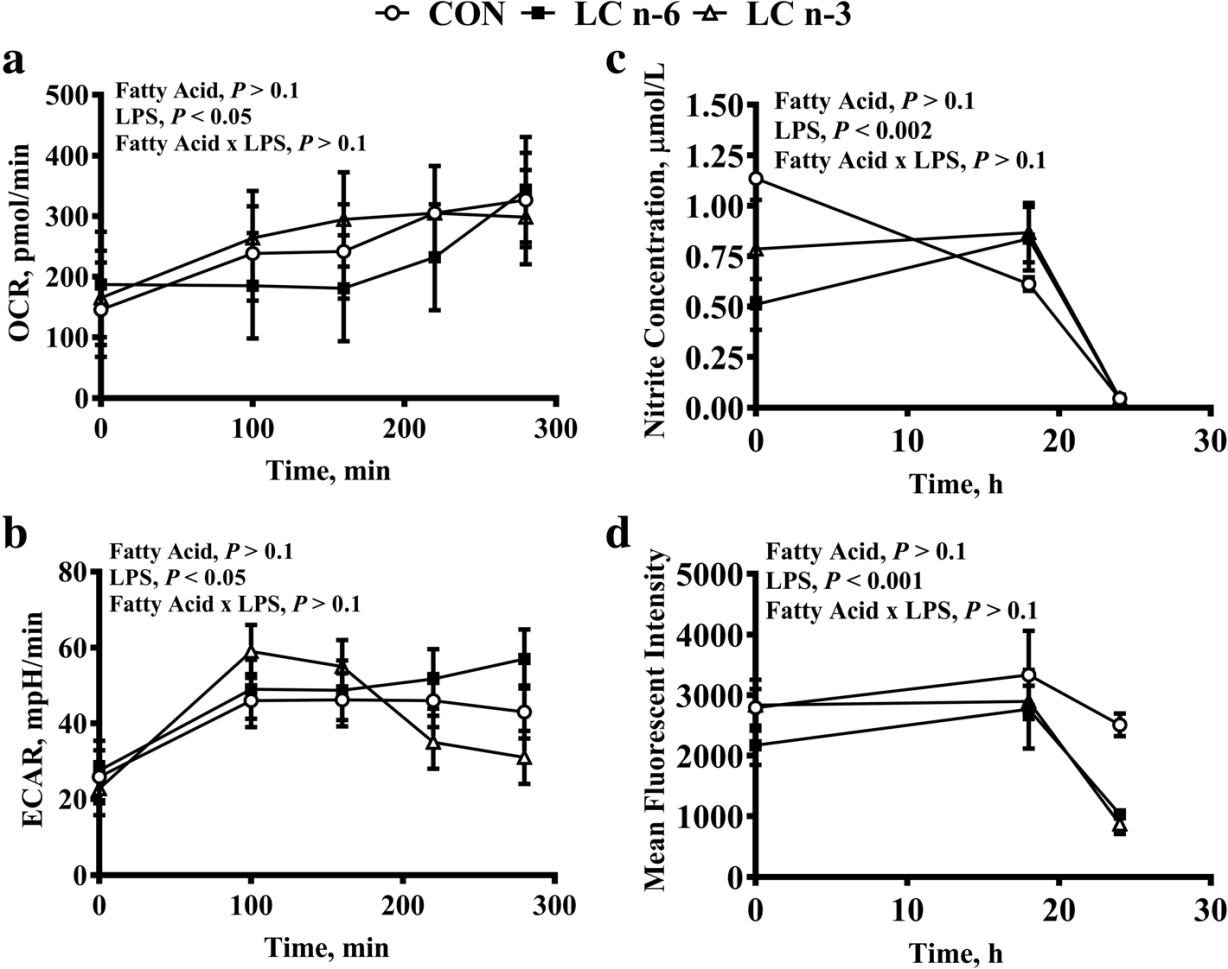
LPS stimulation in porcine alveolar macrophages alters oxidative burst and cellular stress regardless of long chain PUFA enrichment. Representative trace of oxidative burst by measurements of (**a**) OCR, (**b**) ECAR and cellular stress by measurement of (**c**) NO and (**d**) cytoplasmic oxidation in PAM isolated from piglets fed milk replacer with varying fatty acid composition. Measurements were made during culture in absence (Basal) or presence of LPS at varying time points over the course of 24 h. Fatty acid effect, LPS effect and fatty acid-LPS interaction were assessed. Values are least square means ± SEM, *n* = 5. ECAR, extracellular acidification rate; NO, nitric oxide; OCR, oxygen consumption rate

To evaluate oxidative burst in PAM, oxygen consumption rate (OCR) and extracellular acidification rate (ECAR) were evaluated. OCR and ECAR are reported as the average from basal reading (0 to 40 min) compared to hourly measurements taken post LPS stimulation over the course of 4.5 h, which was the maximum cells could withstand being outside of a humidified CO_2_ incubator. Oxygen consumption rate increased over time following LPS stimulation (Fig. 4a). Consistent with an increase in OCR, an LPS effect was observed in the ECAR (Fig. 4b) among all dietary treatments. Changes in NO production and cytoplasmic oxidation, which are indicators of oxidative stress, increased after 18 h then declined after 24 h of LPS stimulation although no dietary effect was detected (Fig. 4c and d).

## Discussion

For the past 40 years, fatty acids have been known to play a role in the immune system and a great deal of research has been conducted to explore their mechanisms of action [5, 49–52]. With the increasing awareness of chronic inflammatory diseases dietary LC-PUFA as modulators for inflammation, particularly the n-3 class, has been an area of research [51, 53–57]. Supplementation with EPA has proven beneficial [58–61], although it has been reported to hinder proper immunological responses against respiratory pathogens [28–30, 62], suggesting its supplementation could be detrimental in cases where acute inflammation is vital [63, 64]. Accordingly, it is important to explore the potential benefits of LC n-6 PUFA supplementation, especially in piglets where the immune system is not fully matured. The aim of our research was to determine if the duality of ARA-derived eicosanoid roles in both inflammatory responses and initiation of resolution could be observed in acute LPS-stimulated PAM.

Previous studies have indicated that supplementation with LC-PUFA can enrich PUFA content in immune cells, thus altering immune cell function [7, 22, 27, 65–67]. Additionally, maternal PUFA supplementation was demonstrated to alter immune cell PUFA content and eicosanoid production in offspring [25, 68, 69]. Our results demonstrate that 21 d is a sufficient duration to effectively enrich the fatty acid content of alveolar macrophages in milk formula-fed neonates. Increasing dietary long chain n-6 PUFA resulted in ARA enrichment being 2-fold higher in PAM compared to CON. However, higher EPA concentrations from LC n-3 fed pigs abated ARA enrichment in PAM by more than 50% (Table 1). In macrophages, supplementation with LC-PUFA leads to incorporation in both the phospholipid membrane and neutral lipid fractions with the highest incorporation found in the phospholipid membrane [22, 70]. This is important because stimulation of toll-like receptor 4 (TLR-4) from LPS activates cytosolic phospholipase A_2_ (cPLA_2_) for the release of ARA from the phospholipid membrane, which is associated with increased COX-2 and PGE_2_ expression [17, 50, 71–73]. As such, we expected increased cellular ARA content to increase *COX-2* mRNA abundance and subsequently PGE_2_ production upon LPS stimulation. Long chain n-6 enriched PAM had higher abundance of *COX-2* mRNA and were 2-fold higher in PGE_2_ secretion upon LPS stimulation (Fig. 2). These findings are consistent with previous work from Fritsche et al. [25] and Møller et al. [67] in which alveolar macrophages from pigs receiving fish oil for 28 d either maternally-supplied or supplemented demonstrated decreased ARA content and PGE_2_ production in PAM following LPS stimulation.

Early stages of immune cell stimulation promote endogenous 5-LOX to oxidize ARA for LT synthesis leading to the transcription and production of pro-inflammatory cytokines [74]. In this study, the abundance of *ALOX5* (gene for 5-LOX) was significantly lower following LPS stimulation in LC n-6 PAM compared to CON and LC n-3 PAM. Alveolar macrophages from the LC n-3 treatment group displayed the highest level of *ALOX5* mRNA abundance (Fig. 2a), although *TNF*-α abundance was highest in PAM from the LC n-6 treatment group post-LPS induction (Fig. 2b). Long chain n-3 PUFA, such as EPA, can be metabolized by 5-LOX to produce less potent pro-inflammatory mediators [75]. This in part could explain why higher levels of *ALOX5* mRNA is observed in the LC n-3 group, but *TNF-α* mRNA is not. Previous studies have demonstrated that supplementation with EPA significantly lowers *TNF*-α mRNA abundance in macrophages [26, 63]; although other studies indicate mRNA abundance to be unaltered and secretion to be lowered with EPA treatment [76]. Macrophages from mice fed diets containing fish oil more than 7% higher than our study demonstrated lowered *TNF*-α mRNA abundance and secretion at 6 weeks of age when stimulated with 5 μg/mL LPS for 12 h and lowered even further after 15 weeks of supplementation [26]. It is possible that TNF-α is regulated post-transcriptionally in a time and dose dependent manner. While secretion of TNF-α was not evaluated in this study, our results do demonstrate a positive effect on *TNF*-α mRNA abundance of LC-PUFA enriched PAM, with LC n-6 enrichment having the strongest effect. While the mRNA abundance of *IL-6* was lower in CON macrophages post LPS-stimulation, no detectable difference was observed in PAM from the LC n-6 or n-3 groups (Fig. 2c). In alveolar macrophages from weaned pigs fed high n-6 (5% sunflower oil) or n-3 (5% fish oil) diets, no detectable difference in IL-6 production was observed between the groups following LPS stimulation [67]. Studies have suggested that TNF-α and IL-6 negatively regulate one another during innate immune responses. In human peripheral blood mononuclear cells (PBMC), IL-6 suppressed TNF-α abundance and production [77]. Similar effects were observed following gram-positive infection in mice. In TNF-α^−/−^ mice, increased levels of IL-6 were observed after inoculation with *Rhodococcus aurantiacus,* but was decreased following TNF-α administration [78]. Enrichment from a LC n-6 diet may provide an opposite effect in which high dietary levels of ARA promote elevated *TNF*-α abundance and subsequently downregulate the abundance of *IL-6* similar to effects observed in LC n-3 dietary treatments.

Several studies have demonstrated the immunosuppressive capabilities of PGE_2_ in varying innate and adaptive immune cells attributed to lipid-mediator class switching [12, 18, 79, 80]. While the abundance of *IL-10* was not elevated following LPS stimulation (Fig. 3b) in our study, this could be because the abundance of *TNF*-*α*, a very potent pro-inflammatory cytokine, is still elevated and elevation in *IL-10* occurs downstream of the initial lipid-mediator class switch. It is important to note that there were lower *ALOX5* abundance and higher *ALOX12/15* mRNA abundance in LC n-6 PAM compared to CON and LC n-3 PAM after LPS stimulation (Fig. 2a). This suggests that the initial stages of a lipid-mediator class switch have occurred within a 24-h time period post macrophage stimulation. This assumption is supported by the work of Levy et al. in which human polymorphonuclear neutrophils (PMN) exposed to PGE_2_ in vitro then challenged with fMLP (N-formylmethionine-leucyl-phenylalanine) switched from 99% 5-LOX activity to 87% 15-LOX activity within 5 h [81]. Mice injected with TNF-α had rapid increases in leukotriene B_4_ within an hour of injection, then a drastic decrease back to baseline as lipoxin A_4_ levels spiked after 4 h [81].

As expected, an increase in OCR and ECAR were observed upon LPS stimulation, however a dietary effect was not observed (Fig. 4a and b). Immune cells stimulated with LPS, should enhance the nuclear translocation of the transcription factor NFκB, thus activating genes for the generation of reactive oxygen (ROS) and nitrogen species (RNS). Excessive RNS and ROS production can be deleterious [6]. While an LPS effect on OCR and ECAR were observed up to 4.5 h post LPS stimulation, we further investigated if dietary long chain n-6 PUFA had an effect on oxidative burst at later time points within a 24-h period. As such, we evaluated the production of NO and cytoplasmic oxidation at 18 and 24 h post LPS stimulation. Despite the increase in OCR and ECAR, we observed a transient increase in NO production and cytoplasmic oxidation following LPS treatment after 18 h, however a dietary effect was not detected. By 24 h post LPS stimulation a decline in NO production from all dietary treatments were observed. Both n-6 and n-3 PUFA supplementation have been reported to both enhance and suppress NO production in macrophages [82–84]. In vitro studies with murine macrophages have demonstrated the same conflicting results. In J774 cells, lower concentrations of both ARA and EPA (5 μmol/L) increased NO production 48 h post LPS stimulation (2.5 μg/mL) compared to higher doses of ARA and EPA (100 μmol/L) [85]. Contrary to these findings, RAW264 cells stimulated with 50–100 μmol/L EPA reduced NO production 24 h post LPS stimulation (0.15 μg/mL) compared to ARA stimulated cells [86]. In neutrophil-like cells (HL-60) increased oxidative burst activity was observed with both ARA and EPA treatment in a dose and time dependent manner [87]. It is evident that ARA and EPA effects on the generation of ROS and RNS species can be dose and time dependent, but they can also be cell dependent. The mechanism behind these discrepancies requires further evaluation. Additional measures of oxidative stress merit further investigation. It remains to be determined if sow milk can be sufficiently enriched in LC n-6 PUFA to benefit the offspring. There is conflicting evidence on the potential benefits and side effects of LC n-3 PUFA supplementation in pigs [88, 89]. Equally, increased LC n-6 PUFA supplementation could impact litter size, feed intake, growth rate and survivability. As such, further work to investigate the impact on overall health in pigs when fed increased LC n-6 PUFA is warranted.

## Conclusions

Taken together, these data suggest that supplementing neonatal pig diet with LC n-6 PUFA, preformed ARA, can enrich 20:4n-6 content in PAM and lead to higher *COX-2* mRNA abundance, PGE_2_ production, and pro-inflammatory cytokine expression upon LPS stimulation. Furthermore, macrophage activation with LPS increases *ALOX12/15* mRNA and lowers *ALOX5* abundance in LC n-6 enriched PAM signifying that within a 24-h period, the initial stages of a lipid-mediator class switching has ensued. Increased dietary long chain n-6 PUFA could be an effective means for enhancing a stronger, well-balanced response to respiratory challenges in neonatal pigs. We recognize the inherent limitations of in situ studies, and further investigation is warranted to further assess the benefits of the dual nature of PGE_2_ signaling within the host. Further work also is needed to examine long-term safety, clinical efficacy and economic viability.

## Additional file

Additional file 1: Validation of PAM isolation procedure. (PDF 285 kb)

## Abbreviations

ARA: Arachidonic acid
COX: Cyclooxygenase
cPLA_2_: Cytosolic phospholipase A_2_
ECAR: Extracellular acidification rate
ELISA: Enzyme-linked immunosorbent assay
EPA: Eicosapentaenoic acid
fMLP: N-formylmethionine-leucyl-phenylalanine
FSC: Forward scatter
GC-MS: Gas chromatography-mass spectrometry
h: Hours
HI-FBS: Heat inactivated- fetal bovine serum
IL: Interleukin
LC: Long chain
LOX: Lipoxygenase
LT: Leukotriene
LX: Lipoxin
MFI: Median fluorescent intensity
NCBI: National Center for Biotechnology Information
NO: Nitric oxide
OCR: Oxygen consumption rate
PAM: Porcine alveolar macrophages
PBMC: Peripheral blood mononuclear cells
PMN: Polymorphonuclear neutrophils
PUFA: Polyunsaturated Fatty Acids
RNS: Reactive nitrogen species
ROS: Reactive oxygen species
RPL: Ribosomal protein L
SDHA: Succinate Dehydrogenase complex subunit A
SSC: Side scatter
TLR: Toll-like receptor
TNF-α: Tumor necrosis factor-alpha
YWHAZ: Tyrosine 3-monooxygenase/tryptophan 5-monooxygenase activation protein zeta polypeptide
