# Supplementary material for: Dietary arachidonate in milk replacer triggers dual benefits of PGE2 signaling in LPS-challenged piglet alveolar macrophages
Source: J Anim Sci Biotechnol. 2019 Feb 15;10:13. doi: 10.1186/s40104-019-0321-1 (PMC6376662; doi:10.1186/s40104-019-0321-1)
Supplement: Supplementary file 1 — Validation of PAM isolation procedure. (PDF 285 kb) [file 40104_2019_321_MOESM1_ESM.pdf]

Additional File 1

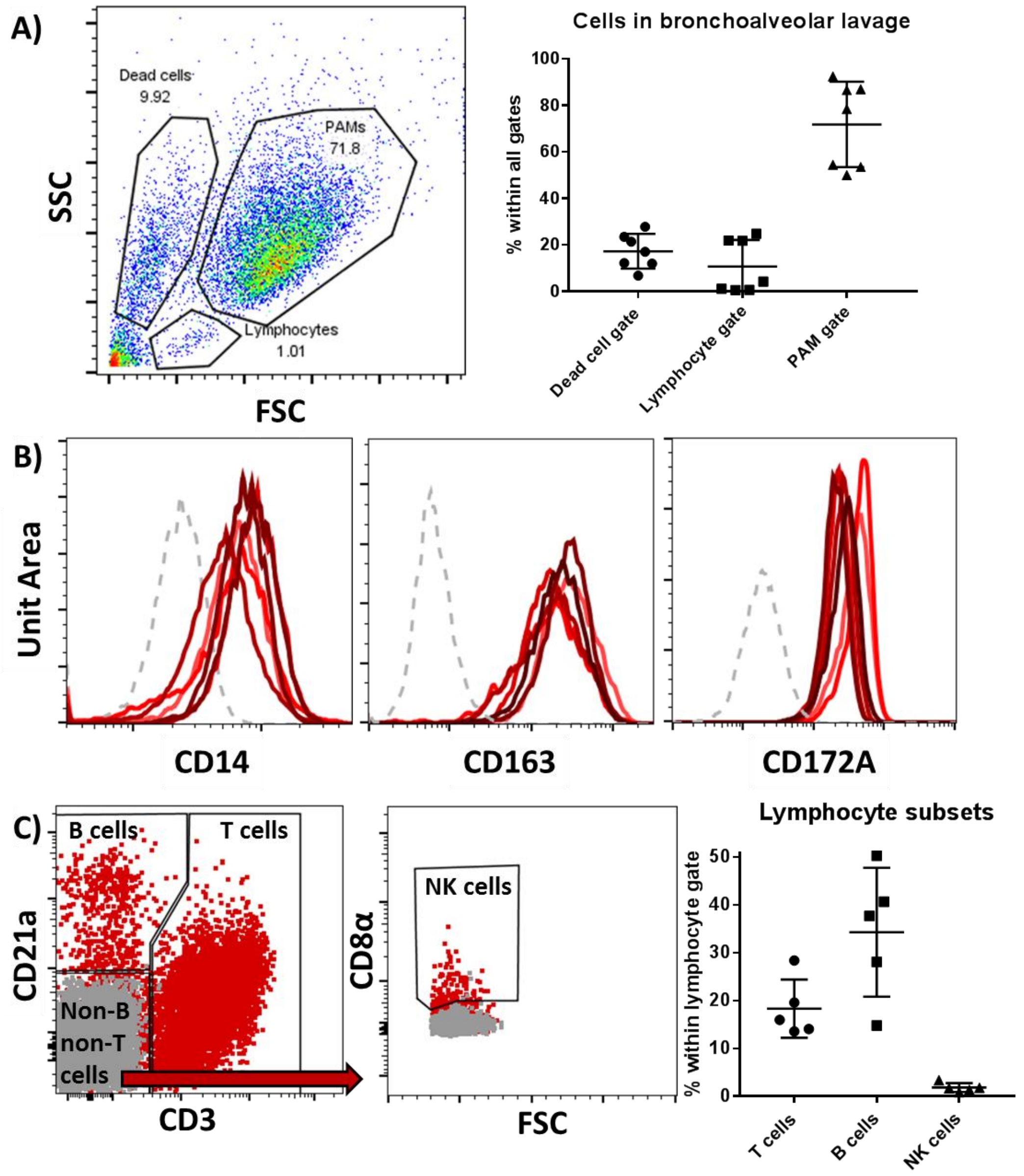

Additional figure 1: Validation of PAM isolation procedure. The phenotype and survival of cells isolated by alveolar lavage was analyzed via flow cytometry. A) Left: FSC/SSC characteristics were used to distinguish dead cells, PAM cells and lymphocytes. Right: Percentage of cells in the different FSC/SSC gates. In order to exclude debris from the analysis, 100% represents the sum of frequencies in all three gates (Dead cells + PAMs + Lymphocytes). B) Within the PAM FSC/SSC gate, all cells exhibited the characteristic $CD14^{low}CD163^{+}CD172A^{+}$ expression pattern of PAM. Grey dashed lines represent isotype controls, red solid lines represent staining of cells isolated from six animals. C) T cells, B cells and NK cells were distinguished via CD3, CD21a, and CD8α expression analysis within the Lymphocytes gate. On average 18.3% were T cells, 34.3% B cells and 1.8% NK cells.
